# The role of biofilms as environmental reservoirs of antibiotic resistance

**DOI:** 10.3389/fmicb.2015.01216

**Published:** 2015-10-31

**Authors:** José L. Balcázar, Jéssica Subirats, Carles M. Borrego

**Affiliations:** ^1^Catalan Institute for Water ResearchGirona, Spain; ^2^Group of Molecular Microbial Ecology, Institute of Aquatic Ecology, University of GironaGirona, Spain

**Keywords:** aquatic ecosystems, biofilms, mobile genetic elements, antibiotic resistance genes, aquatic resistome

## Abstract

Antibiotic resistance has become a significant and growing threat to public and environmental health. To face this problem both at local and global scales, a better understanding of the sources and mechanisms that contribute to the emergence and spread of antibiotic resistance is required. Recent studies demonstrate that aquatic ecosystems are reservoirs of resistant bacteria and antibiotic resistance genes as well as potential conduits for their transmission to human pathogens. Despite the wealth of information about antibiotic pollution and its effect on the aquatic microbial resistome, the contribution of environmental biofilms to the acquisition and spread of antibiotic resistance has not been fully explored in aquatic systems. Biofilms are structured multicellular communities embedded in a self-produced extracellular matrix that acts as a barrier to antibiotic diffusion. High population densities and proximity of cells in biofilms also increases the chances for genetic exchange among bacterial species converting biofilms in hot spots of antibiotic resistance. This review focuses on the potential effect of antibiotic pollution on biofilm microbial communities, with special emphasis on ecological and evolutionary processes underlying acquired resistance to these compounds.

## Environmental Biofilms

Nature is often unpleasant. It is then better to face environmental uncertainties under the principle of “strength through unity". In many habitats, either natural or artificial, microorganisms attach themselves to surfaces, either abiotic or biotic, forming a complex matrix of biopolymers known as biofilm that protect them from environmental hazards (Costerton et al., 1978). Biofilms may be composed of a single bacterial species (e.g., *Vibrio cholerae*, Teschler et al., 2015) but more frequently they are formed by a complex and diverse community of microorganisms (bacteria, algae, fungi and protozoa) embedded in an extracellular matrix of polysaccharides, exudates, and detritus (Costerton et al., 1978; Wimpenny et al., 2000). Many microbial species are able to change their lifestyle (free-living vs. attached) depending on their physiological status and the physicochemical conditions in their surroundings, taking advantage of the greater availability of organic matter in suspended particles and surfaces (Simon et al., 2002; Grossart et al., 2004; Grossart, 2010; Teschler et al., 2015). In aquatic habitats, biofilms develop not only in benthic substrata, such as streambed cobbles and sand (epilithic and epipsammic biofilms, respectively), but also on floating macro– and microaggregates (Simon et al., 2002). From an ecological perspective, microorganisms in environmental biofilms actively participate in organic matter decomposition, nutrient dynamics and biogeochemical cycling, being a key component of cosystem functioning (Sabater and Romaní, 1996; Sabater et al., 2002; Simon et al., 2002; Battin et al., 2007; Romaní, 2010). Moreover, streambed biofilms are considered as good indicators of the overall water quality and the ecological status of the system (i.e., ecosystem health) (Burns and Ryder, 2001; Sabater et al., 2007). It is then of special interest to assess how biofilm communities respond to anthropogenic pollution of aquatic environments (e.g., rivers, lakes, and reservoirs) considering the increasing amount of chemical compounds (metals, personal care products and drugs used in veterinary and human medicine) released into these waterbodies mainly through wastewater treatment plant (WWTP) eﬄuents and agricultural run-off (Pruden et al., 2006; Sarmah et al., 2006; Baquero et al., 2008). This review focuses on the role of streambed biofilms as reservoirs of antibiotic resistant bacteria and resistance genes, providing a general overview of the causes and consequences of a chronic exposure of biofilm communities to sub-inhibitory concentrations of antibiotics and their role in the spread and persistence of antibiotic resistance.

## Biofilms And Antibiotics

Biofilms show an increased survival and resistance to environmental and chemical stressors (e.g., antibiotics) mainly, but not only, by the protection conferred by the extracellular polysaccharide matrix (Mah and O’Toole, 2001; Stewart and Costerton, 2001; Donlan, 2002; Donlan and Costerton, 2002; Stewart, 2002; Hall-Stoodley et al., 2004; Høiby et al., 2010). In biofilms, bacterial cells exhibit 10 to 1,000 times less susceptibility to specific antimicrobial agents compared with their planktonic counterparts (Gilbert et al., 2002). This reduced susceptibility is caused by a combination of different factors, namely: (i) a poor antibiotic penetration into the polysaccharide matrix; (ii) the arbitrary presence of cells showing a resistant phenotype (known as “persisters”); and (iii) the presence of either non-growing cells or cells that triggered stress responses under unfavorable chemical conditions within the biofilm matrix (Stewart and Costerton, 2001; Stewart, 2002). These protective mechanisms act synergistically to those responsible for conventional resistance linked to the presence of antibiotic resistance genes (ARGs) in bacterial genomes or extrachromosomal elements, yielding an overall increased resistance of biofilms to antimicrobial compounds. For instance, β-lactamase producing bacteria offered increased protection in biofilms because the β-lactam antibiotic, such as ampicillin, was inactivated by those β-lactamases (Anderl et al., 2000). Moreover, the *ampC* gene of *Pseudomonas aeruginosa* biofilms was strongly induced by exposure to antibiotics, such as imipenem (Bagge et al., 2004). Additionally, biofilm formation may result as a defensive reaction to the presence of antibiotics. Hoffman et al. (2005) found that sub-inhibitory concentrations of aminoglycosides induce biofilm formation as part of a defense response in *Escherichia coli* and *P. aeruginosa*. Similar results were described by Salcedo et al. (2014), who observed that sub-inhibitory concentrations of tetracycline and cephradine induce biofilm formation and enhance the transfer rate of the pB10 plasmid among the biofilm biomass (*E. coli* and *P. aeruginosa*) at rates 2–5 times faster than without antibiotic treatment. Since biofilm formation is also common for most bacterial pathogens, the enhanced resistance of biofilms to antibiotics is a serious concern for human health as many chronic infections are linked to biofilm growth on either natural surfaces (e.g., teeth, lungs) or foreign-body devices (e.g., pacemakers, catheters, prosthetic heart valves). The characteristics, composition, growth dynamics, and resistance mechanisms of clinically relevant biofilms have been reviewed in detail by several authors (Donlan and Costerton, 2002; Parsek and Singh, 2003; Hall-Stoodley et al., 2004; Høiby et al., 2010), and are out of the scope of this review. In clear contrast, lesser is known about the role of environmental biofilms as natural reservoirs of ARGs, their contribution to ARGs spreading among biofilm inhabitants and their transfer to free-living bacteria, increasing the risk for their transmission to aquatic microorganisms and potential human pathogens (Vaz-Moreira et al., 2014 and references therein).

## Environmental Biofilms Under Chemical Stress

Many aquatic systems (rivers, lakes, reservoirs) are affected by human activities such as continuous discharges from WWTP eﬄuents. Under such conditions, macro- and microorganisms inhabiting these waterbodies are exposed to a low but constant concentration of a wide range of chemical pollutants (antibiotics but also analgesics, anti-inflammatory, and psychiatric drugs, β-blockers, pesticides, etc.) that alter their behavior at different levels, with consequences that we are only beginning to grasp (Bernier and Surette, 2013; Boxall, 2014). Several studies have demonstrated the effects of the so-called emerging pollutants on the composition, activity, and resilience of streambed biofilms (Bonnineau et al., 2010; Ricart et al., 2010; Proia et al., 2011, 2013a,b; Osorio et al., 2014), although the ecological implications of such background pollution are difficult to envisage. A serious drawback arises when comparing the environmental concentrations of antibiotics measured in polluted aquatic habitats (from ng/L to μg/L) to those used to treat bacterial infections (i.e., therapeutic concentrations, which are usually ≥1 mg/L). Since environmental concentrations of antimicrobial compounds are several orders of magnitude below the minimum inhibitory concentration (MIC) of most bacterial pathogens, their antibiotic effect is doubtful, if any (Waksman, 1961; Davies, 2006; Davies et al., 2006; Davies and Davies, 2010). Current data strongly suggest that antibiotics, at these sub-MIC concentrations, act as signaling molecules mediating a wide variety of cell processes (gene transcription and expression, quorum sensing, inter- or intra-species communication, biofilm formation, among others; Davies, 2006; Romero et al., 2011; Sengupta et al., 2013; Andersson and Hughes, 2014), instead of causing growth arrest or cell death. Moreover, low concentration of antibiotics may also trigger different stress responses that might accelerate horizontal gene transfer (HGT) and the spread of ARGs in a broad range of bacterial species (Beaber et al., 2004; Miller et al., 2004; Maiques et al., 2006). Under this perspective, the chronic exposure to subinhibitory antibiotic concentrations that occurs in most aquatic ecosystems offers new avenues for research that deserve exploration. For instance, is the effect of this chronic exposure strong enough to shape the composition of microbial communities? Or is it buffered by the many other physico-chemical constraints that microbes face in their habitat? Is the antibiotic pollution adding a background noise that interferes with normal communication among bacterial cells in their habitats (e.g., biofilms)? If so, how can this noise effect be measured? And what about activity? Does antibiotic pollution have measurable effects on biogeochemical cycles at both local and global scales? In this regard, Roose-Amsaleg and Laverman (2015) have recently reviewed 31 articles dealing with the effects of antibiotics on microorganisms involved in biogeochemical cycles to ascertain if environmental concentrations of these compounds have side-effects on such cycles, with special focus on N cycling (anammox, denitrification, and nitrification). Despite the few studies available and the variability in terms of antibiotic types and conditions tested, conclusions of their work point to a clear alteration of microbial activity in key biogeochemical cycles, thus affecting ecosystem functioning at different levels.

Despite these considerations, it is now clear that chronic exposure to antibiotics, even at very low concentrations, promotes and maintains a pool of resistance genes in natural microbial communities (Séveno et al., 2002; Allen et al., 2010; Sengupta et al., 2013; Andersson and Hughes, 2014). It should be mentioned, however, that most of these genes, although conferring a resistant phenotype when expressed, are probably not “true” resistance genes (Martinez et al., 2015) thus having a function distantly related to that under therapeutic conditions (Allen et al., 2010; Martinez et al., 2015). Notwithstanding this, current data indicate that the extensive use of antibiotics over the last century has generated a selective pressure that has accelerated the acquisition and spread of ARGs among environmental bacteria posing a risk for human health assuming the striking capacity of microbes to share genes.

## Acquisition And Spread Of Args In Biofilms

Susceptible bacteria may become resistant to antibiotics through chromosomal mutations or by HGT, being the latter the major contributor to the spread of antibiotic resistance determinants. The significance of HGT to microbial adaptation was initially recognized when antibiotic-resistant pathogens were identified (Sobecky and Hazen, 2009). HGT is mediated by mobile genetic elements (MGEs), which play an important role in the evolution and adaptation of bacterial species to new and/or changing environmental conditions (Frost et al., 2005). MGEs are segments of DNA encoding a variety of enzymes and proteins that mediate their movement within the host genome (intracellular mobility) or between bacterial cells (intercellular mobility). Interchange of DNA fragments between a cell donor and a receptor takes place through conjugation, transformation, or transduction, whereas intracellular movement is facilitated by integrons and transposons (Modi et al., 2014).

Together with phage transduction and natural transformation, the exchange of genetic material through conjugation is one of the most efficient pathways to disseminate antibiotic resistance among bacterial cells, where donor and recipient cells are in close contact. Conjugation is mainly mediated by the so-called “conjugative plasmids”, although “conjugative transposons” are also capable of triggering the process. One of the most important aspects of conjugative plasmids is that they can be exchanged among both related and phylogenetically distant bacteria (Dionisio et al., 2002). The high cell density and close contact among cells within the biofilm matrix together with increased genetic competence and accumulation of MGEs in these habitats convert them into an optimal scenario for the acquisition and spread of ARGs (Fux et al., 2005). Several studies have shown increased conjugation efficiencies in biofilms when compared to free-living bacterial cells. In fact, conjugation of the broad-host-range plasmid RP4 between two species of *Pseudomonas* occurred in a biofilm reactor at high frequencies (Ehlers and Bouwer, 1999). *In situ* assessment of gene transfer rates in biofilms using automated confocal laser scanning microscopy revealed conjugation rates 1,000-fold higher than those determined by classical plating techniques (Hausner and Wuertz, 1999). Molin and Tolker-Nielsen (2003) also showed that the efficiency of gene transfer seems to be correlated with the biofilm surface, suggesting that a high surface/volume ratios favor transfer within or between biofilm populations.

The diversity and abundance of ARGs in environmental biofilms have been investigated by several authors to unveil differences in the concentration of target genes between planktonic and benthic compartments. Less information is available, however, on the contribution of MGE to the acquisition and spread of ARGs among biofilm inhabitants and between them and free-living bacteria. **Table 1** summarizes some relevant studies dealing with the presence, diversity and abundance of ARGs in biofilms from different environmental settings such as rivers exposed to WWTP eﬄuent discharges, WWTP and drinking water network pipelines, experimental mesocosm, and sand filters. Although not exhaustive, **Table 1** provides a general overview of results obtained by different research groups studying the role of environmental biofilms as hot spots for the accumulation and transfer of ARGs. Schwartz et al. (2003) demonstrated that the *vanA* gene, which confers a high-level resistance to vancomycin, was detected in drinking water biofilms in the absence of any vancomycin-resistant enterococci, suggesting a potential gene transfer from them to autochthonous bacteria in drinking water systems. Gillings et al. (2008) investigated the presence of a MGE, the class 1 integrase (*intI1*) gene, in bacterial isolates collected from diverse environmental samples near Sydney. Authors found that 1 to 3% of bacterial isolates from lake sediments were *intI1* positive, while in biofilms from a groundwater treatment plant, the number of *intI1*-positive isolates reached 30% despite no antibiotics were used as selective agents for culturing. Moreover, Engemann et al. (2008) found that the abundance of six genes conferring resistance to tetracycline was reduced at different rates in the water column, and some genes, particularly *tetW*, readily migrated into biofilms. Transfer to biofilms did not, however, completely explain disappearance of *tet* genes from the planktonic compartment and other factors such as sunlight and potential microbial degradation would probably contributed (Engemann et al., 2006, 2008). In a similar experimental approach but using periodical piglet waste loadings, Zhang et al. (2009) observed that *tet* genes migrate rapidly to biofilms, where they persist longer than in adjacent waters. Recently, Farkas et al. (2013) also observed that 9.4% of isolates from drinking water biofilms harbored class 1 integrons, which were mainly detected in bacteria (e.g., *Enterobacteriaceae*) that may be associated with microbiological contamination.

**Table 1:** Studies on antibiotic resistance and related genes in environmental biofilms.

Type of biofilm	Sampling Point^†^	Target ARG	Organism	Method	Pollution source^†^	Main findings	Reference
River bed Wastewater pipeline	DWN HWP Upstream WWTP WWTP eﬄuent	*vanA mecA ampC*	Enterococci Staphylococci *Enterobacteriaceae*	Cultivation, PCR	UWW HWW	• All target genes were amplified from hospital wastewater biofilms.	Schwartz et al., 2003
						•*van*A and *amp*C genes were detected in all wastewater biofilms.	
GWTP	GACF	*Intl1*	Multi-species biofilm	PCR, CE-SSCP	GWP	• In biofilms from the groundwater treatment plant, the number of *intl1*-positive colonies reached 30%.	Gillings et al., 2008
Experimental Mesocosms	Peripheral biofilms grown in mesocosm	*tetO, tetW, tetM, tetQ, tetB* and *tetL*	Multi-species biofilm	qPCR	CWS	• *tet(W)* gene showed the highest migration from the water column to biofilms.	Engemann et al., 2008	
						• Only 15% of ARGs disappearance rate was caused by migration to biofilms.
River bed Wastewater pipes	WWTP HWP	*aac*(6’)-*Ie* +*aph*(2”) *mecA, tetA, tetB*	Multi-species biofilm	qPCR, PCR	UWW HWW	• The highest concentration of all genes was observed in the hospital pipeline.	Börjesson et al., 2009
Experimental mesocosms	Peripheral biofilms grown in mesocosm	*tet(O), tet(W), tet(M), tet(Q), tet(B)* and *tet(L)*	Multi-species biofilm	qPCR	PWS (periodic pulse addition)	• Studied genes migrate rapidly from water to biofilms, where they persisted longer than in adjacent water.	Zhang et al., 2009
Horizontal subsurface constructed wetland	Influent Wetland biofilm Eﬄuent	*tetA, tetB, tetM, sul1, ermB, ampC, qnrS*	Multi-species biofilm	qPCR	UWW	• All genes were detected in the three studied compartments	Nõlvak et al., 2013
						• ARGs concentration in the biofilm and in the eﬄuent were affected by system operational parameters.
Drinking water treatment plant	Clarifier sand filter	*Intl1, sul1 qacEΔ1*	Multi-species biofilm	PCR	UWW	• All class 1 integron genes detected were positive for the *qacEΔ1*gene. In turn, only 37.5% of class I integron genes were positive for *sul1*.	Farkas et al., 2013
River bed	Upstream river waters WWTP discharge Downstream river waters	*qnrA, qnrB, qnrS bla_TEM_, bla_CTX-M_, bla_SHV_ ermB, sulI, sulII, tetO, tetW*	Multi-species biofilm	qPCR	UWW	• Relative abundance of target ARG’s was significantly higher in biofilm samples collected downstream the WWTP discharge point than in biofilms collected in upstream waters.	Marti et al., 2013
River bed	Six sites along the river (Upstream-downstream)	*vanA, vanB * aacA-aphD, *mecA ermA, ermB tetA, tetB, tetK, tetM*	Multi-species biofilm	PCR	LF	• Only three antibiotic resistance genes (ARG) were detected within the 147 samples collected.	Winkworth, 2013
River bed	Upstream river waters WWTP discharge Downstream river waters	*qnrA, qnrB, qnrS aac*(6’)-*Ib-cr*	Multi-species biofilm	PCR	UWW	• The *qnr*S gene was the most prevalent among *qnr* genes in the environment.	Marti et al., 2014b


*^†^WW, Wastewater; WWTP, Wastewater treatment plant; GWTP, Groundwater treatment plant; DWN, Drinking water network; HWP, Hospital wastewater pipeline; HWW, Hospital wastewater; UWW Urban wastewater; CWS, cattle waste slurry; LF, Livestock farming; PWS, Piglet waste slurry; GACF, Granulated activated charcoal filter; GWP, Groundwater pollution; CE-SSCP, Capillary electrophoresis single strand conformation polymorphism.*

Because biofilms play an important role as reservoirs for ARGs, they could be considered as biological indicators of antibiotic resistance pollution in the same way as river ecologists use streambed biofilms as indicators of the overall “ecological status” of the river ecosystem (Sabater et al., 2007). The chronic exposure to sub-MIC concentration of antibiotics exerts a selective pressure on biofilm bacterial communities that may stimulate the emergence and spread of antibiotic resistance (Allen et al., 2010; Andersson and Hughes, 2014; Marti et al., 2014a; Chow et al., 2015). The presence of other pollutants, such as heavy metals from feed additives, organic, and inorganic fertilizers, pesticides and anti-fouling products, also contributes in the co-selection of antibiotic resistance because the close location of genes encoding for these resistance phenotypes in the same MGE (Seiler and Berendonk, 2012). Such exposures may eventually have consequences on the selection and abundance of MGEs, thereby facilitating the spread of ARGs among different species; different biofilm compartments (e.g., epilithic, epipsammic, and hyporheic streambed); or even between different prokaryotic communities as recently assessed by plasmid metagenomics (Sentchilo et al., 2013). Besides, several studies provided evidence that ARGs tend to accumulate in biofilms rather than in the planktonic compartment. In this regard, Börjesson et al. (2009) found a high proportion of genes encoding resistance to aminoglycosides and tetracyclines in biofilm samples collected at a WWTP. Winkworth (2013) demonstrated that, while the levels of ARGs in biofilm samples collected along the Taieri River were low, sites subjected to combined influences of greater human activity and intensive dairy farming showed an increased level of ARGs. Likewise, a study carried out by our research group clearly showed the effect of WWTP eﬄuents on the prevalence of several ARGs in the Ter River, accompanied by a significant increase in their relative abundance in biofilm samples collected downstream the WWTP discharge point (Marti et al., 2013). Moreover, we have investigated the prevalence of plasmid-mediated quinolone resistance (PMQR) determinants in ciprofloxacin-resistant strains isolated in biofilm and sediments from a WWTP discharge point and its receiving river (upstream and downstream sites). We observed that, while the number of strains harboring PMQR determinants was higher in sediments, PMQR-positive strains were also detected in biofilm samples, especially in those from the WWTP discharge point and downstream sites (Marti et al., 2014b). In a study carried out in a horizontal subsurface flow constructed wetland, Nõlvak et al. (2013) found that copy numbers of *tetA* and *sul1* genes in the wetland biofilms were one order of magnitude higher than in the eﬄuent water, despite the fact that this facility had a similar efficiency to conventional WWTP in removing ARGs from wastewater. Altogether, these studies undoubtedly demonstrate the contribution of biofilms in the acquisition and spread of ARGs.

## Antibiotic Resistance In Biofilms Assessed By Metagenomics

Until the last decade our knowledge of antibiotic resistance has largely depended on data provided by traditional culture-based methods (Cockerill, 1999). Although useful, these data are limited and biased towards cultivable members of the community. Recent advances in genomics and metagenomics are now providing new avenues for understanding evolutionary processes controlling antibiotic resistance mechanisms and their spreading among microbial populations.

To date, several thousand metagenomes have already been sequenced from a large variety of environments, and this number is set to grow rapidly in the forthcoming years. Most of these metagenomes are publically available through various databases and annotation platforms, such as MG-RAST (Meyer et al., 2008), CAMERA (Sun et al., 2011), and IMG/M (Markowitz et al., 2012), which provide additional insight in the function of complex microbial communities through comparative analyses. Moreover, the availability of specialized databases such as the ARG Database (ARDB; Liu and Pop, 2009), the Comprehensive Antibiotic Resistance Database (CARD; McArthur et al., 2013), the Integron Database (INTEGRALL; Moura et al., 2009), the Bush, Palzkill, and Jacoby’s collection of curated β-lactamase proteins (http://www.lahey.org/Studies/), and the implementation of high-throughput sequence analysis tools such as BLAT (Kent, 2002), USEARCH (Edgar, 2010), and DIAMOND (Buchfink et al., 2015), provide a comprehensive molecular toolbox that allow a better understanding of the evolution, ecology, and spread of antibiotic resistance in different organisms and ecosystems.

**FIGURE 1:**
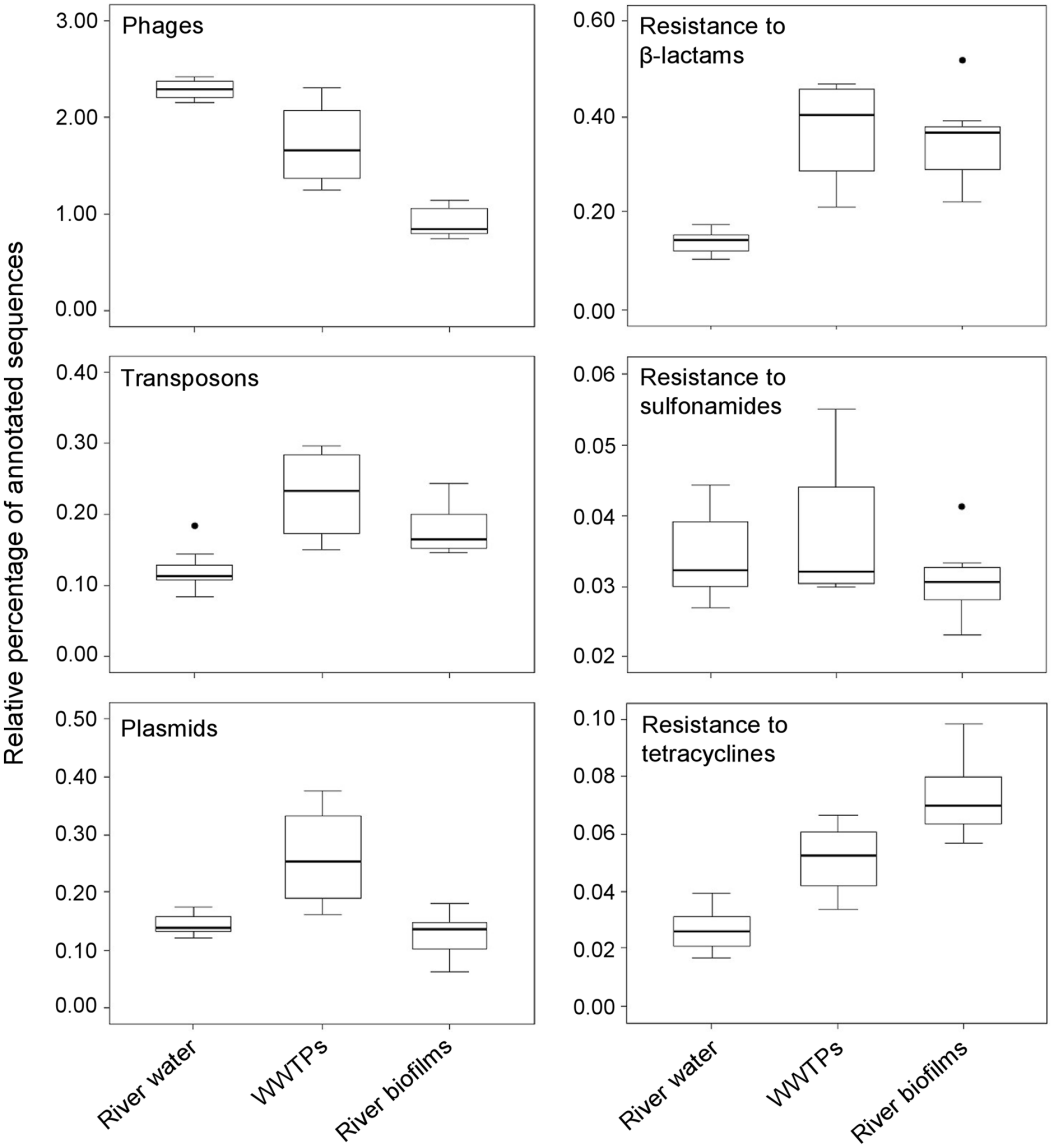
**Metagenomic exploration of the resistome from environmental sources.** Relative distribution of reads assigned to six functional subsystems among 23 metagenomes (based on MG-RAST annotation, *E*-value = 10^-5^) Data are normalized by the total annotated sequences and are expressed as a percentage. The horizontal line in each box plot represents the mean of the relative distribution in each of the three environments (river water, WWTPs, and river biofilms), and the black circles represent the outliers. The 23 metagenomes used for the analysis are available at http: //metagenomics.anl.gov. Accession numbers for river waters: 4511251.3, 4511252.3, 4511253.3, 4511254.3, 4511255.3, 4511256.3, and 4511257.3; WWTPs: 4455295.3, 4463936.3, 4467420.3, and 4511199.3; and river biofilms: 4528142.3, 4528143.3, 4528144.3, 4528145.3, 4528146.3, 4528147.3, 4589537.3, 4589538.3, 4589539.3, 4589540.3, 4589541.3, and 4589542.3.

We have conducted a comparative analysis of selected metagenomes corresponding to several projects and environments publically available in the MG-RAST database (http://metagenomics.anl.gov/) to provide an overall insight on the prevalence of MGEs and ARGs in environmental biofilms. This analysis showed that MGEs-related sequences, such those from phages and plasmids, were found in a lower proportion in metagenomes from river biofilms than those from WWTPs and river water environments. Remarkably, transposons were detected in a higher proportion in WWTPs and river biofilms than those from river water environments (**Figure 1**). Similarly, sequences related to genes conferring resistance to β-lactam antibiotics were also detected more frequently among microbial communities from WWTPs and streambed river biofilms than those from river water environments. Sequences related to genes conferring resistance to tetracyclines were also abundant in WWTPs and river biofilms, but to a lesser extent than β-lactams. Finally, no differences in the proportion of genes conferring resistance to sulfonamides were observed among the examined environments.

Interestingly, the analysis of the selected metagenomes also showed that two acid mine drainage biofilm samples from the Richmond Mine (4441138.3 and 4441137.3) yielded a high proportion of sequences related to genes conferring resistance to β-lactam antibiotics (5.7 to 7.2%). These relatively high values of β-lactamases might be related to the higher proportion of transposons in these acidophilic biofilms (0.5 to 1.6%) than those detected in environments close to neutral pH such as riverbed biofilms, WWTPs and freshwater systems (**Figure 1**).

A recent study revealed a remarkable abundance and diversity of genes encoding transposases in the metagenome of a hydrothermal chimney biofilm (Brazelton and Baross, 2009). The comparative analysis between this metagenome (4461585.3) and the metagenomes mentioned above confirmed these observations (8.1% of transposase sequences), but similar proportions were observed for β-lactamases between the hydrothermal vent biofilms and those from river water environments. The high relative proportion of transposases may favor an enhanced gene transfer between bacterial genomes that confer new and useful accessory functions, including resistance to heavy metals or antimicrobial compounds. The presence of genes conferring resistance to β-lactams in environments not subjected to antibiotic pollution such as deep sea vents or pristine systems raises interesting questions not only about the origin and ecological function of these genes in nature but also the criteria that researchers adopt when defining a resistance gene (Martinez et al., 2015).

## Final Remarks And Future Prospects

Biofilms occur in almost any submerged surface in both natural and man-made systems providing a suitable and optimal environment for the growth, activity, and interaction of different bacterial species. Biofilms also provide a shelter where to cope with transient or permanent stress conditions, also favoring metabolic interactions and genetic interchange between different bacterial species struggling for survival in a changing environment. Punctual or continuous discharges of pharmaceutical compounds into aquatic systems might constitute not only a selective pressure on aquatic bacterial communities that stimulate the transmission and spread of ARG, but also a chronic source of background biochemical noise that may potentially interfere the communication networks that microbes finely tuned during evolution. Although little information is available on the actual capacity of aquatic bacteria to transfer antibiotic-resistance determinants to potential human pathogens, current data corroborate that environmental biofilms are true reservoirs of ARGs. Further research is needed; however, to elucidate to which extent such hot spots of antibiotic resistance may constitute a serious concern for human health, how the diversity and abundance of ARG change between different biofilm compartments, how this resistance genetic pool moves among communities and how this gene transfer varies in response to the amount of chemical pollution (antibiotics but also other stressors such as heavy metals and xenobiotic compounds) in the receiving waters. The continuous refinement of sequencing technologies (e.g., metagenomics, metatranscriptomics) and bioinformatic tools and the availability of specialized and properly curated databases may help to reach these goals and hit new research targets. Answering these (and other) questions will provide a better knowledge of the transfer dynamics of resistance genes at ecosystem level (between species, communities, and/or habitats), yielding clues to fight against antibiotic resistance and the threat that it poses to the environment and to the human health.

## Conflict of Interest Statement

The authors declare that the research was conducted in the absence of any commercial or financial relationships that could be construed as a potential conflict of interest.
